# Urine Proteins Identified by Two-Dimensional Differential Gel Electrophoresis Facilitate the Differential Diagnoses of Scrapie

**DOI:** 10.1371/journal.pone.0064044

**Published:** 2013-05-21

**Authors:** Lise Lamoureux, Sharon L. R. Simon, Margot Plews, Viola Ruddat, Simone Brunet, Catherine Graham, Stefanie Czub, J. David Knox

**Affiliations:** 1 Prion Laboratory Services Section, Public Health Agency of Canada, Winnipeg, Manitoba, Canada; 2 National Centres for Animal Disease, Canadian Food Inspection Agency, Lethbridge, Alberta, Canada; 3 GE Healthcare, San Francisco, California, United States of America; 4 Department of Medical Microbiology, University of Manitoba, Winnipeg, Manitoba, Canada; University of Maryland School of Medicine, United States of America

## Abstract

The difficulty in developing a diagnostic assay for Creutzfeldt - Jakob disease (CJD) and other transmissible spongiform encephalopathies (TSEs) stems in part from the fact that the infectious agent is an aberrantly folded form of an endogenous cellular protein. This precludes the use of the powerful gene based technologies currently applied to the direct detection of other infectious agents. To circumvent this problem our research objective has been to identify a set of proteins exhibiting characteristic differential abundance in response to TSE infection. The objective of the present study was to assess the disease specificity of differentially abundant urine proteins able to identify scrapie infected mice. Two-dimensional differential gel electrophoresis was used to analyze longitudinal collections of urine samples from both prion-infected mice and a transgenic mouse model of Alzheimer's disease. The introduction of fluorescent dyes, that allow multiple samples to be co-resolved and visualized on one two dimensional gel, have increased the accuracy of this methodology for the discovery of robust protein biomarkers for disease. The accuracy of a small panel of differentially abundant proteins to correctly classify an independent naïve sample set was determined. The results demonstrated that at the time of clinical presentation the differential abundance of urine proteins were capable of identifying the prion infected mice with 87% sensitivity and 93% specificity. The identity of the diagnostic differentially abundant proteins was investigated by mass spectrometry.

## Introduction

Transmissible spongiform encephalopathy (TSE) diseases are untreatable, uniformly fatal degenerative syndromes of the central nervous system that can be transmitted both within as well as between species. The infectious agent is generally recognised as a misfolded isoform of the host encoded prion protein. Propagation occurs through a not as yet understood posttranslational process, resulting in the conversion of cellular prion protein, PrPc, into the misfolded disease associated isoform, PrPd [1], [2]. An accumulation of PrPd in the central nervous system coincides with disease progression and consequently definitive diagnostic tests rely upon the detection of the disease associated isoform in brain tissue post-mortem.

While high concentrations of PrPd accumulate in the CNS, the detection of the low amounts of PrPd present in other tissues makes ante mortem tests based on the presence of the etiologic agent problematic. One solution to this challenge has been the development of cell free techniques that exploit the ability of PrPd to seed the conformational conversion of a PrPc substrate [3], [4]. Similar to the amplification of mRNA by PCR this procedure results in the generation of greatly increased amounts of PrPd facilitating subsequent detection by traditional immunologic methods. One of the more recent iterations of such an assay is real-time quaking-induced conversion (RT-QuIC) [5], [6]. In RT-QuIC recombinant PrP is used as the substrate and the dye thioflavin T is added to the reaction mixture. The binding of thioflavin T to aggregates of PrPd causes a change in its emission spectrum that can be monitored in real time.

Analysis of CSF samples from patients with suspected CJD has demonstrated RT-QuIC to be both highly sensitive and specific [7], [8]. Thus, in a clinical context the addition of RT-QuIC would improve the differential diagnosis of neurodegenerative diseases with similar presentation. Despite this success the invasive nature of a CSF based test is not suitable for large scale screening of an asymptomatic population. Thus RT-QuIC is unable to address concerns regarding the prevalence of subclinical infections that could compromise the safety of surgical instruments or the blood supply. In these instances an ante-mortem TSE test based on a matrix or body fluid that would permit easy access and be amenable to repeated sampling is required. Despite the demonstration that plasma samples from preclinical hamsters infected with scrapie can be identified using an enhanced RT-QuIC protocol no non-invasive ante mortem test has yet been validated [9].

An alternative approach to the development of ante-mortem tests focussed on PrP detection has been to identify host encoded proteins whose abundance, in an easily accessible tissue or body fluid, is modulated in a characteristic fashion in response to TSE infection. Altered protein profiles in CSF in response to TSE infection have been observed, however, due to the aforementioned reasons CSF is not a suitable sample for large scale screening [10]–[15]. Previously, we demonstrated that the differential abundance of a panel of proteins found in urine was sufficient to identify BSE infected cattle at the clinical stage of the disease with a high degree of accuracy irrespective of the presence of confounding factors such as breed, gender or age [16]. To extend these findings we have used scrapie infected mice and a transgenic mouse model of Alzheimer's disease to identify prion disease specific proteins exhibiting differential abundance in urine. The resulting biomarkers demonstrated sensitivity and specificity similar to that of CSF samples analyzed using RT-QuIC when applied to a naïve sample set.

## Materials and Methods

### Ethics Statement

This study was carried out in strict accordance of the guidelines of the Canadian Council on Animal Care. The protocols were approved by the Animal Care Committee of the Canadian Science Centre for Human and Animal Health (permit numbers: H-10-006, H-11-004). All surgery was performed under inhaled isoflurane anaesthesia, and all efforts were made to minimize suffering.

### Scrapie Mice

Female C57BL/6 mice were intracerebrally infected with a 1% brain homogenate of scrapie strain ME-7 prepared as previously. Control mice were inoculated with a prion free 1% brain homogenate in PBS. The first clinical sign of prion disease in this scrapie mouse model was the cessation of nesting which started approximately 16 weeks post infection. Following the cessation of nesting the mice progressed rapidly to terminal disease [17].

Chilled urine was collected overnight by placing individual mice in modified metabolic cages with free access to water but withholding food to avoid contamination. The same biological replicates (4 infected and 4 control animals) were used at 2 week intervals over the course of the disease. This generated 8 samples at each of the 9 time points (wk1–wk17). All urine was stored at −80C until processing. Thawed samples were concentrated and dialyzed by rinsing in Amicon 5K MWCO filtration units with 3×1 ml PBS. Samples were concentrated to a volume of not more than 250 µl then soluble protein was extracted and purified using GE Healthcare's 2D Clean up kit, and quantified with the Plus One 2D quant kit [18].

Urine for glomerular filtration rate (GFR) determination was collected as above. Mice were weighed when placed in metabolic cages and following the 16 hr overnight urine collection. Urine was stored at −80°C until use. Blood from the same mice was collected the following morning by heart puncture under inhaled isoflurane anaesthesia into 2 mL lithium heparin Vacuette tubes (Grenier Bio-one #454237). The blood was separated by centrifugation at 5000× g for 30 minutes with the plasma layer moved to fresh 1.5 ml microfuge tubes for storage at −80°C until use. The IDEXX Vettest instrument was used to measure creatinine levels and total protein in both the blood and the urine with the appropriate chip chemistries (Urine P∶C Ratio or CREA and TP. IDEXX Laboratories, Markham ON). Testing with each chip was done as per manufacturers recommendations.

### Alzheimer's Mice

The transgenic Alzheimer's disease model(B6SJL-Tg(APPSwFlLon, PSEN1*M146L*L286V)6799Vas/J, JL #006554) originated from a C57BL/6 * SJL background and has been reported to display a steady increase of markers of neurological disease starting at 10 weeks of age that become marked by 28 weeks of age. Terminal stage disease for this transgenic model occurred at 40–44 weeks and matched endpoints reported previously for females of this genotype [19]. The control mice were produced by crossing a C57BL/6J female with a SJL/J male. The F1 control mice are heterozygous for B6 and SJL alleles at all loci in their genome and mice used in this study were female. The same biological replicates (4 affected and 4 control animals) were used at 2 week intervals over the course of the disease. This generated 18 time points of which 9 monthly sets of 8 samples were used. All urine was collected in chilled adaptors for metabolic cages and stored at −80°C until processing. Protein extraction and quantitation was performed as described above [17].

### 2D DIGE

Two dimensional difference in gel technology (2D-DIGE) was employed for the differential protein expression analyses. The main attributes of DIGE are the capability to resolve multiple samples on one gel, and the use of an internal standard for cross gel normalization. The internal standard is critical for the removal of gel to gel variation, accurate quantitation and reproducibility [20]. The internal gel standard was created by pooling equivalent amounts of protein from biotypes of scrapie and Alzheimer's disease as well as their corresponding controls at each time point. This pooled sample was labelled with Cy2 minimal dye while infected, diseased and control samples were randomly labelled within each time point with either Cy3 or Cy5 minimal label to avoid dye bias as per [18]. Samples were resolved on 24 cm Immobiline drystrips pH 4–7 and 12–18% Tris-based pre-cast gels (Jule Inc., Milford, CT)

### Gel image acquisition

Gels were scanned by a Typhoon 9410 Variable Mode Imager (GE HealthCare). Photomultiplier tube (PMT) voltage settings were adjusted to maximize utilization of the dynamic range of the imager. Despite the large dynamic range of the imager (10^5^), the presence of major urinary proteins (MUPs) in milligram per millilitre quantities in mouse urine posed a problem [21]. PMT settings that provided higher signal resolution at the low end, where small changes in signal are more critical, also resulted in signal saturation at the high end. It was decided to let the MUP proteins, which clustered in the PI 4.5/25 kDa range, to saturate. The saturated spots were then manually excluded in the DeCyder™ Differential In-gel Analysis (DIA) module and not copied into DeCyder™ Biologic Variation Analysis (BVA) module during the import of DIA gel images. The exclusion of the saturated spots allowed quantitative analysis of the remaining proteins.

Scrapie samples were resolved on 34 gels. Each gel was comprised of the internal standard and two biological samples. From the 34 gels, 68 gel images each representing a biological replicate were acquired for analysis. The acquired gel images were analyzed using the BVA and DeCyder™ Extended Data Analysis (EDA) software modules. This resulted in the detection, quantification, and matching of an average of 1330 of the 1938 features detected on the master gel across the 34 scrapie gels, normalized by the internal standard.

Alzheimer's samples were resolved on 36 gels. Each gel was comprised of the internal standard and two biological samples. From the 36 gels run 67 gel images of suitable quality, each representing a biological replicate were acquired for analysis. Using the scrapie Master Gel as a template DeCyder™ DIA and BVA analysis resulted in the detection, quantification, and matching of an average of 1655 of the 1938 master features across the 36 Alzheimer gels, all normalized by the same internal standard.

### Principle Component Analysis (PCA)

In DeCyder™ PCA is used as a descriptive and exploratory tool. PCA is essentially an unsupervised method for reducing the dimension of the variables in a multidimensional space by describing all features selected by a single eigenvalue. After the PCA analysis, one tries to interpret the first few principal components in terms of the original variables, and thereby get a greater understanding of the data. To reproduce the total system variability of the original p variables, all principle components are needed. However, the 1^st^ component will represent the largest source if the variability, the 2^nd^ component, the next largest, and so forth. Hence, if the first few PCs account for a large proportion of the variability (80–90%), the objective of dimension reduction is achieved. The PCA implementation in DeCyder™ uses the NIPALS algorithm for calculation of the principal components since it is not as memory demanding as other algorithms such as Single Value Decomposition (SVD) [22].

PCA analyses were used to generate an overview of the relationship between spots maps where each spot map represents the spotmap features of a urine sample obtained from a particular mouse at a particular time point. In most cases, the features selected correspond to all spots matched among ≥80% of the gels and exhibiting statistically significant (ANOVA p≤0.01) changes in abundance. Corresponding PCA analyses were performed to examine whether any of the proteins were outliers. In this instance each dot represents a protein. The further from the origin a dot is, the larger the variance in calculated abundance between the spot as matched between gels. The circle on the plot represents a 95% confidence interval and any dot falling outside this circle is considered an outlier. As an outlier could be either a mismatched spot, or a protein that varied dramatically in expression between different samples, dots that fell outside this interval were checked in BVA to make sure that they were properly matched and had the characteristics of a true protein. If they were correctly matched, these outliers can potentially help in the identification of biomarkers.

### Spot Picking

Features of interest were manually excised using a Gilson P1000 Pipetman from silver stained preparative gels and stored in 1% acetic acid. The ART pipet tips were cut with a razor blade to increase the pore size. Silver stained gel cubes were destained with a 1∶1 solution of 30 mM Potassium Ferric cyanide/100 mM Sodium Thiosulfate in water and washed with water and 200 mM Ammonium Bicarbonate for 1 hour prior to reduction (10 mM dithiothreitol, Sigma) 30 minutes at room temperature and alkylation (100 mM iodoacetamide, Sigma) 30 minutes at room temperature. Modified sequencing grade porcine trypsin solution 30 µL (20 ng/µL, Promega, Madison,WI) was added to the gel slice enzyme/protein ratio 1∶50 and then digested for 16 h at 37°C. The peptides were extracted out of the gel slices with 400 µL (50/40/10 v/v acetonitrile/water/formic acid). The sample was then dried by speed vac and stored at −80°C until analysis.

### LC-MS/MS analysis

Peptide mixtures were separated by on-line reversed phase chromatography using a Thermo Scientific EASY-nLC II system with a reversed-phase pre-column Magic C-18AQ (100 µm I.D., 2 cm length, 5 µm, 100 Å,, Michrom BioResources Inc, Auburn, CA) pre-column and a reversed phase nano-analytical column Magic C-18AQ (75 µm I.D., 15 cm length, 5 µm, 100 Å, Michrom BioResources Inc, Auburn, CA) both prepared in-house at the University of Victoria Genome BC Proteomics Centre, at a flow rate of 300 nl/min. The chromatography system was coupled to an LTQ Orbitrap Velos mass spectrometer equipped with a Nanospray II source (Thermo Fisher Scientific). Solvents were A: 2% Acetonitrile, 0.1% Formic acid; B: 90% Acetonitrile, 0.1%Formic acid. After a 249 bar (∼5 µL) pre-column equilibration and 249 bar(∼8 µL) nanocolumn equilibration, samples were separated by a 55 minute gradient (0 min: 5%B; 45 min: 45%B; 2 min: 80%B; 8 min: 80%B).

The LTQ Orbitrap Velos (Thermo Fisher Scientific, Bremen, Germany) parameters were as follows: Nano-electrospray ion source with spray voltage 2.2 kV, capillary temperature 225°C. Survey MS1 scan m/z range 400–2000 profile mode, resolution 60,000 @400m/z with AGC target 1E6, and one microscan with maximum inject time 200 ms. Lock mass Siloxane 445.120024 for internal calibration with preview mode for FTMS master scans: on, injection waveforms: on, monoisotopic precursor selection: on; rejection of charge state:1. The fifteen most intense ions charge state 2–4 exceeding 5000 counts were selected for CID ion trap MSMS fragmentation (ITMS scans 2–16) and detection in centroid mode. Dynamic exclusion settings were: repeat count: 2; repeat duration: 15 seconds; exclusion list size: 500; exclusion duration: 60 seconds with a 10 ppm mass window. The CID activation isolation window was: 2 Da; AGC target: 1E4; maximum inject time: 25 ms; activation time: 10 ms; activation Q: 0.250; and normalized collision energy 35%.

### Data Analysis Parameters

Peak lists were generated from LTQ Orbitrap Velos raw files using the Thermo Finnigan LCQ/DECA Raw file import filter option implemented in Mascot Daemon v2.3 (Matrix Science London). For the Mascot (2.4.1) search of the Mouse IPI_v3.85 database using trypsin digestion, parent ion mass tolerance was set to 20 ppm and fragment ion tolerance was set to 0.5 Da. One miss cleavage was allowed. Carbamidomethylation was set as a fixed modification while methionine oxidation and acetyl-n-terminus were set as variable modifications. Resulting .dat files were loaded into Scaffold 3.6.5 (Proteome Software, Portland, OR) with independent analysis settings. Peptides were accepted if they exhibited a greater than 80% probability under Peptide Prophet. Proteins were considered identified if they exhibited greater than 99% probability under protein prophet and additionally included 2 or more peptides. With these settings, the protein false discovery rate was <0.1%. Proteins containing similar peptides were grouped according to parsimony.

## Results

### Gel image analysis of urine proteome of scrapie infected mice

Multivariate analyses of protein expression data derived from the BVA were performed using the DeCyder™ Extended Data Analysis Software (EDA). EDA is designed to identify the smallest subset of features able to accurately discriminate between experimental groups. The 4 scrapie infected and 4 control mice provided 8 biological samples at each of the 9 time points throughout the course of the disease. In total the 4 control and 4 infected mice generated 34 control and 34 infected usable gel images respectively. Power analysis for 2D-DIGE has demonstrated statistical power of >0.8 for detecting 2-fold changes at p≤0.01 with 4 biological replicates [23], [24].

Spots on a 2D gel may or may not represent individual proteins. To make this distinction gel spots are referred to as features until an associated protein is identified. The data were initially filtered so that only the 135 features present in ≥80% of the gels and exhibiting statistically significant (ANOVA p≤0.01) changes in abundance were considered. Regularized discriminant analysis (RDA) and principle component analysis (PCA) were used to generate an overview of the ability of the 135 features to discriminate between the two experimental groups. Each data point in the PCA analysis represents the set of all 135 features based on variance. Hence, two data points in close proximity to one another indicates that the selected 135 features are very similar in the two samples. A subset of features, able to discriminate accurately between control and infected samples over the entire course of the disease, was not found. Nonetheless, the analysis suggested that a classifier might emerge as the disease evolved. To test this hypothesis infected and age matched samples from earlier time points in the disease course were progressively removed from the analyses.

**Figure 1:**
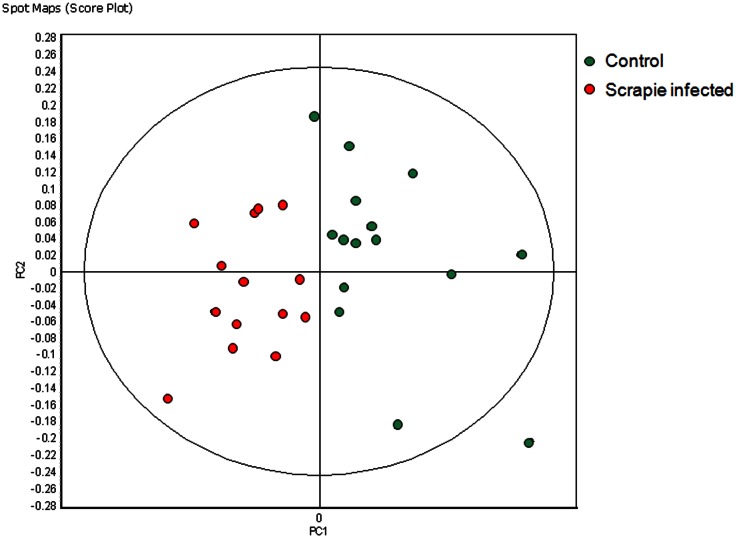
Principle Component analysis of the 14 scrapie infected (red) and 15 control samples (green) collected from 4 control and 4 infected mice at 4 time points: 11, 13, 15 and 17 weeks post infection (wpi). Samples not collected were one infected sample at both 11 and 15 wpi and one control sample at 13 wpi. A classifier based on 2 features was able to correctly classify the 29 samples of the training set with 100% accuracy. (PC1 = 87.4, PC2 = 12.6).

**Figure 2:**
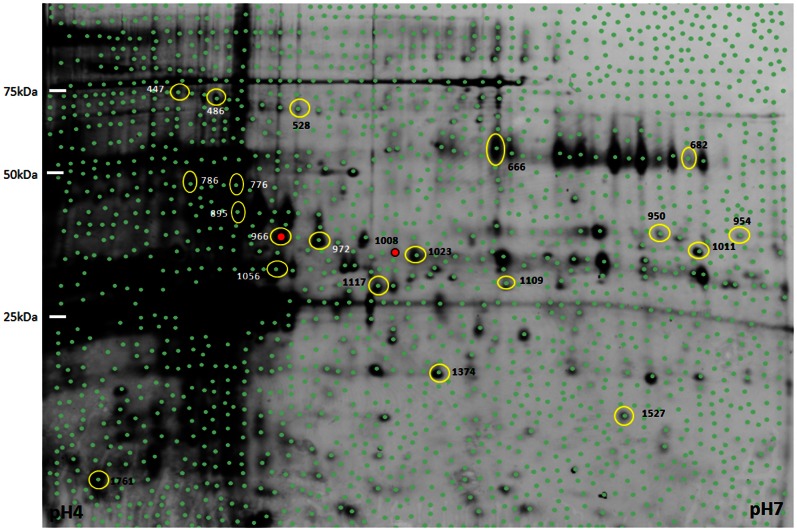
Representative Cy-2 labelled internal standard gel image illustrating the proteins resolved in the pH 4 to pH 7 range. Each green dot represents a feature identified by the DeCyder software. The absence of green dots in the PI 4.5/25 kDa range indicates the location of saturated features that were excluded from the analysis. The Disease Discriminant Classifier was constructed from the 20 features circled in yellow. The positions of the two features utilized to generate the PCA plot in Figure 1 and create a classifier able to discriminate between scrapie infected and age matched control mice with 100% accuracy from 11 weeks post-infection onward are marked with a red dot.

After the removal of each time point, an overview of the new set of features present in ≥80% of the remaining gel images and exhibiting statistically significant (ANOVA p≤0.01) changes in abundance was generated by RDA and PCA. Following the removal of the fifth time point 79 features were present in ≥80% of the 29 remaining gel images and exhibited statistically significant changes in abundance (ANOVA p≤0.01). PCA based on these 79 features segregated the two groups into different quadrants of the PCA chart and the RDA calculated accuracy of a classifier containing the 79 features was 100%. To identify the smallest subset of the 79 features best able to discriminate between control and infected samples, the 79 features were searched by the forward selection (FS) and RDA algorithms. An RDA generated disease specific classifier, based on two identified features, was able to correctly classify the 15 control and 14 infected samples with 100% accuracy. The two features were also used to calculate a PCA of the 14 biological replicates of infected and 15 biological replicates of control samples illustrated in Figure 1. The positions of the two features used in these calculations are illustrated in Figure 2.

### Gel image analysis of urine proteome of a transgenic Alzheimer's mouse model

Multivariate analyses of protein expression data derived from the Alzheimer's disease BVA were performed using the DeCyder™ EDA Software. The biological samples were grouped according to disease state resulting in two groups each representing either 34 samples produced by the diseased mice or 33 samples produced by age matched controls.

The data was filtered so that only the 133 features present in ≥80% of the gels and exhibiting statistically significant (ANOVA p≤0.01) changes in abundance were considered. Discriminant analysis and PCA, were used to generate an overview of the capacity of the 133 features to discriminate between the two experimental groups. This analysis demonstrated that a classifier based on the 133 features was able to discriminate between control and infected samples over the entire disease course with a high degree of accuracy (95.7%±6.4). Classifier subsets, generated by discriminant analysis based on the FS and RDA, containing fewer than 133 features had decreased accuracy. It is important to note that in this and subsequent analyses, the subset of features considered as part of the classifier was dependent upon the training sets selected.

**Figure 3:**
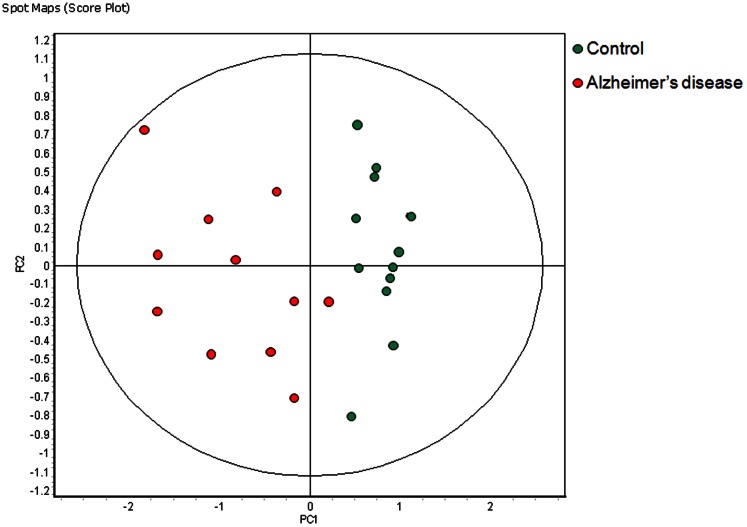
Principle component analysis of the 11 Alzheimer's disease model (red) and the 12 control samples (green) collected from 4 diseased and 4 control mice at 3 time points: 32, 36, and 40 weeks of age. A sample from one of the diseased mice was not obtained at 32 weeks of age. A classifier based on 84 features was able to correctly classify the 23 samples of the training set with 100% accuracy. (PC1 = 62.0, PC2 = 11.9).

To determine whether a classifier with 100% accuracy would emerge as the disease evolved samples from earlier time points in the disease course were progressively removed from the analysis. After the removal of each time point an overview of the new set of features present in ≥80% of the remaining gel images and exhibiting statistically significant (ANOVA p≤0.01) changes in abundance was generated by RDA and PCA. Following the removal of six time points, 84 features were present in ≥80% of the 23 remaining gel images and exhibited statistically significant changes in abundance. PCA based on these 84 features segregated the 11 diseased and 12 control samples into different quadrants of the PCA chart (Figure 3) and RDA calculated the accuracy of the disease specific classifier containing the 84 features to be 100%. Classifier subsets containing fewer than 84 features had decreased accuracy.

Interestingly, 85% of the misclassified samples at earlier time points were generated by one particular diseased mouse and the misclassified samples produced by this mouse occurred at all time points excepting 32 and 36 weeks of age (Table 1).

**Table 1:** Alzheimer's Disease Model Classifiers.

Weeks of Age	# of Spot Maps	# of Proteins	Calculated Accuracy	Misclassified Spot Maps		
9–40	67	133	95.7±6.4	Alz6-13	Alz6-9	AlzCtl15-17
13–40	62	123	96.7±4.6	Alz6-13	Alz4-21	
17–40	54	102	96.0±8.9	Alz6-24	Alz6-21	
21–40	46	92	96.0±8.9	Alz6-24	Alz6-21	
24–40	38	78	97.5±5.6	Alz6-24	Alz6-40	
28–40	30	82	93.3±9.1	Alz6-28		
32–40	23	84	100.0±0			

Classifiers were generated using the features present on 80% of the gels and exhibiting a significant differential abundance (ANOVA, p≤0.01) for the range of samples indicated in the first column. The number of features present in the classifier, the calculated accuracy and the particular samples misclassified are given for each sample set as the earlier time points are progressively removed. The particular mouse is indicated by the number immediately adjacent to the disease indicator and the number separated by a hyphen indicates the age of mouse from which the sample was obtained.

### Identification of Disease Specific Markers

The scrapie model and Alzheimer's disease model BVA results were imported together into EDA. Because the same internal standard Master gel was employed in both BVA analyses the results from the two experiments could be compared directly to one another. The biological samples were grouped according to disease state resulting in three groups. One comprised of the 34 scrapie infected samples; one comprised of the 34 Alzheimer's disease samples; and a third group comprised of both the 34 scrapie control and the 33 Alzheimer's disease control samples.

The data was filtered so that only features present in ≥80% of the gels and exhibiting statistically significant (ANOVA p≤0.01) changes in abundance were considered. This screen resulted in the identification of 284 features. Discriminant analysis and PCA of the 284 features were used to generate an overview of the capacity of the 284 features to discriminate between the three experimental groups. The PCA plot showed that no one group was able to separate itself from the other two and the accuracy calculated by discriminant analysis was 33% (not shown). This indicated that an analysis of the differential abundance of the features did not improve the classification of the samples above what would be expected from a random assignment of the samples to one of the three groups.

**Figure 4:**
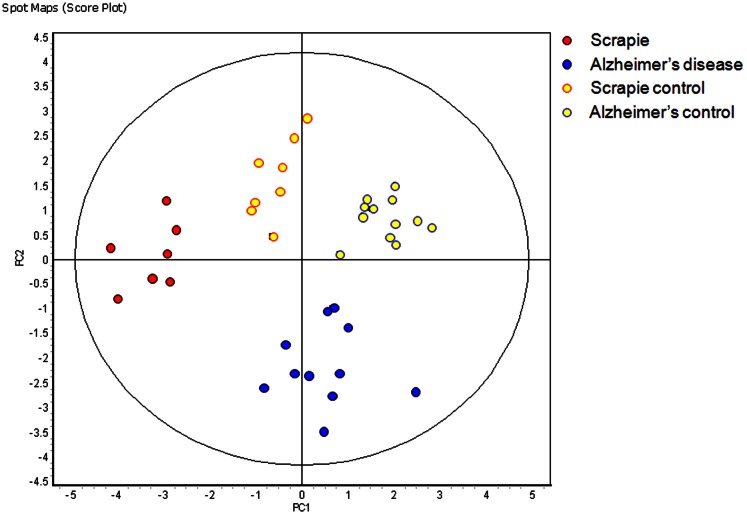
Principle component analysis of the 169 feature classifier applied to the 7 scrapie infected (red) and the 8 corresponding scrapie control samples (yellow with red boarder) obtained from 4 infected and 4 control mice at 15 and 17 weeks post infection combined with the 11 Alzheimer's diseased (blue) and the 12 corresponding Alzheimer's disease control samples (yellow with blue boarder) obtained from 4 diseased and 4 control mice at 32, 36 and 40 weeks of age. (PC1 = 27.9, PC2 = 24.5).

The analysis was then restricted to those samples acquired around the time that the animals started to exhibit clinical signs of the disease. This cohort consisted of the 15 and 17 weeks post infection (wpi) scrapie samples and the 32, 36 and 40 weeks of age Alzheimer's disease samples and their corresponding controls. The data of the new set was filtered so that only the 169 gel features present in ≥80% of the gel images and exhibiting statistically significant (ANOVA p≤0.01) changes in abundance were taken into account. The RDA calculated the accuracy of the disease discriminant classifier, based on the 169 features was 100%. PCA based on the same 169 features revealed that not only was a clear separation of the 7 scrapie and 11 Alzheimer's samples observed, but a clear separation between the 8 scrapie and 12 Alzheimer's disease control samples was also observed (Figure 4).

The disease discriminant classifier generated, comprised of 169 features, appeared to be influenced to a certain extent by confounding factors. To limit the confounding strain bias and the presence of general markers of neurological disease, pairwise comparisons were performed. To identify features reflecting strain differences, only the gel images of the 8 scrapie control and 12 Alzheimer control samples were analyzed. 47 features, exhibiting statistically significant (ANOVA p≤0.01) changes in abundance between the two different mouse strains, were considered strain sensitive features. These 47 strain sensitive features were removed from the 169 features of the disease discriminant classifier reducing the number of features in the disease discriminant classifier to 122.

To identify general markers of neurological disease, two pair wise comparisons were performed. In one, the gel images of the 7 scrapie infected samples and the 8 scrapie control samples were analyzed. This identified 70 disease specific features present on ≥80% of the gels and exhibiting statistically significant (ANOVA, p≤0.01) changes in abundance. Similarly, a pair wise comparison of the gel images of the 11 Alzheimer's disease samples and the 12 Alzheimer's disease control samples identified 75 disease specific features present on ≥80% of the gels and exhibiting statistically significant (ANOVA, p≤0.01) changes in abundance. The 15 features common to both disease specific lists were considered general markers of neurological disease. When the 122 features remaining in the disease discriminant classifier were scrutinized, 5 of the features considered to be general markers of disease were found and removed. The RDA calculated accuracy of the 117 features remaining in the disease discriminant classifier was 100%.

An additional pairwise comparison of the 7 scrapie infected samples and the 11 Alzheimer' disease samples was made. The features present on ≥80% of the gel images and exhibiting differential abundance (ANOVA, p≤0.01) were identified. Selecting features common to this list and the disease discriminant classifier resulted in the selection of 55 of the remaining 117 features of the disease discriminant classifier. These proteins were arguably those best able to discriminate between the control and diseased groups. The RDA calculated accuracy of the 55 features remaining in the disease discriminant classifier to discriminate between the Alzheimer diseased, scrapie infected and the combined control group was 100%.

The gel images of the 55 features remaining in the disease discriminate classifier were manually inspected and only the 20 features deemed sufficiently intense to permit isolation and identification of the associated protein/peptide were selected (Figures 2+5). The RDA calculated accuracy of the 20 feature disease discriminant classifier was 98%±3.7 in classifying the samples of the training set. The 11 Alzheimer's disease and 7 scrapie samples were correctly identified as well as 19 of the 20 control samples. The misclassified sample was an Alzheimer's disease sample at 32 weeks of age. PCA provides a visual overview of this analysis (Figure 6).

**Figure 5:**
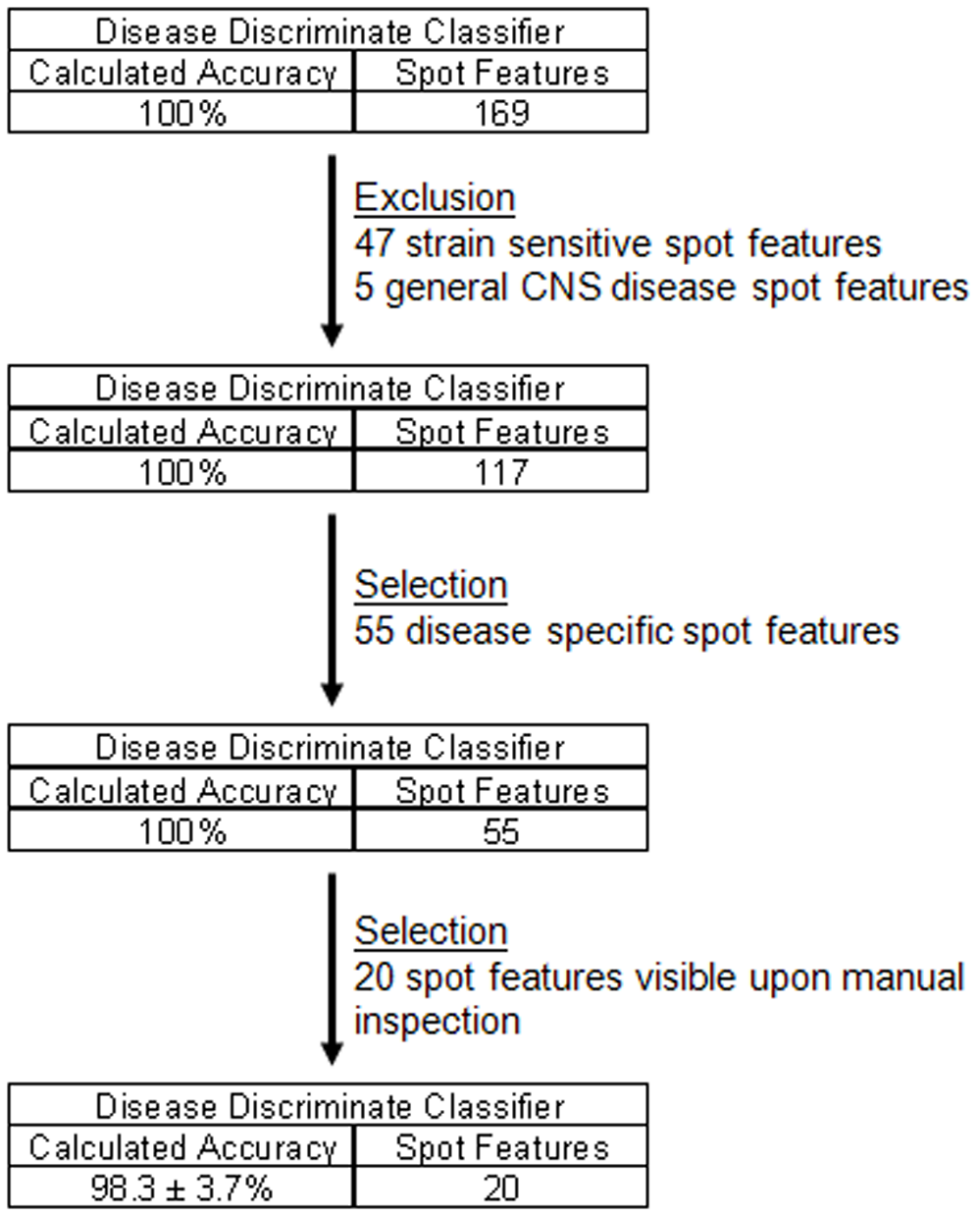
Flow chart depicting the development of the Disease Discriminant Classifier based upon 20 identified proteins from the 169 proteins that were present in 80% of the 38 gel images representing the clinical stage Alzheimer and scrapie sample sets exhibiting significant differential abundance (ANOVA p≤.01).

**Figure 6:**
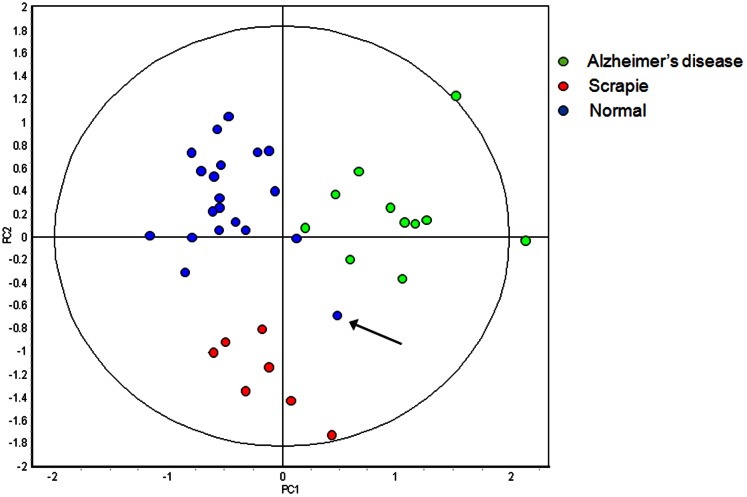
Principle component analysis of 7 scrapie infected samples (red) at 15 and 17 weeks of age, 11 Alzheimer's disease samples (green) at 32, 36 and 40 weeks of age, and the 20 corresponding control samples (blue) when analyzed by the 20 protein Disease Discriminant Classifier. **Samples were produced by 4 scrapie infected mice, 4 Alzheimer diseased mice and the 8 corresponding control mice.** Thirty seven of the 38 samples were correctly classified. The 32 week old control sample, misclassified as having Alzheimer's disease, is identified by an arrow.

Eleven of the 20 features of the disease discriminant classifier excised from a preparative gel were subsequently analysed by an LTQ Orbitrap Velos mass spectrometer (Table 2). The column number heading in Table 2 relates to the similarly numbered feature observed in Figure 2. The greater resolution of the mass spectrometry analysis demonstrated that, despite visually having physical characteristics expected of singular proteins (Figure S1), most features represented a mixture of proteins. A limitation of the mass spectrometry analysis was an inability to determine how much each of the proteins associated with a particular feature contributed to the differential abundance observed. The sensitivity of the mass spectrometry analysis makes contamination an addition potential source of the features apparent complexity. For example, the presence of junction plakoglobin in 10 out of 11 features seems unlikely.

**Table 2:** Mass Spectrometry Analysis.

Identified Protein (22)	Accession #	MW	447		486		776		895		972		1011		1023		1056		1109		1374		1527	
kininogen 2 isoform 2	IPI00857103	48 kDa	12	33%	9	31.0%																		
Protein AMBP	IPI00127352	39 kDa							4	13.0%	6	26.0%					2	6.90%						
Kallikrein-1	IPI00323869	29 kDa							10	50.0%														
Urokinase-type plasminogen activator	IPI00129102	48 kDa											9	26%										
Prostaglandin-H2 D-isomerase	IPI00114252	21 kDa													4	17%								
Deoxyribonuclease-1	IPI00308684	32 kDa					2	5.6%																
Transthyretin	IPI00127560	16 kDa																					6	63%
Napsin-A	IPI00113797	46 kDa															2	12%						
Lymphocyte antigen 6C1	IPI00129134	14 kDa																					3	28%
Insulin receptor-related protein	IPI00123516	145 kDa													2	0.62%								
Peptidyl-prolyl cis-trans isomerase	IPI00987580	18 kDa									2	11.0%												
14-3-3 protein sigma	IPI00118286	28 kDa									4	19.0%												
Pro-epidermal growth factor	IPI00132121	133 kDa													2	2.10%								
Serum albumin	IPI00131695	69 kDa																	3	5.90%				
Major urinary protein 1	IPI00968968	21 kDa			3	20.0%	2	13.0%	7	40.0%							6	38%						
Junction plakoglobin	IPI00229475	82 kDa	2	3.00%	3	21.0%	5	8.7%	2	3.0%	16	26.0%	3	4.60%	3	4.60%	2	3.00%			2	3.00%	2	3.00%
Desmoplakin	IPI00553419	333 kDa			26	9.6%	4	2.3%			11	5.4%					2	0.69%						
Desmoglein-4	IPI00380242	114 kDa									2	1.8%												
Plakophilin-1	IPI00124111	81 kDa			2	3.7%					5	8.5%												
Tubulin alpha-1C chain	IPI00403810	50 kDa									3	9.4%												
Tubulin beta-2A chain	IPI00338039	50 kDa									5	14.0%												
actin, cytoplasmic 2-like	IPI00989022	26 kDa									2	11.0%												

Eleven of the spot features utilised in the disease discriminate classifier and highlighted in Figure 5 were excised and subjected to mass spectrometry analysis. The number heading each column relates to the number assigned to the particular spot features by the DeCyder software. The number of peptides (left subcolumn) and protein coverage (right subcolumn) are denoted beside each identified protein.

Nonetheless, an ongoing question is whether or not proteins identified in urine are indicative of the pathogenesis occurring in the CNS or other organs. For example, in addition to its involvement in neurodegeneration transthyretin as well as napsin A have been associated with renal dysfunction [25]–[31]. To determine if the presence of transthyretin and napsin A in the urine were indicative of renal dysfunction in scrapie infected mice the glomerular filtration rate of scrapie infected mice was measured (Table S1). This demonstrated that at 120 days post infection a significant (p = 0.012) reduction in the glomerular filtration rate of infected mice relative to age matched controls was observed (Table S2).

### Classifier Validation and Characterisation

In order to get a true measure of the accuracy of the 20 features of the disease discriminate classifier, its ability to correctly classify a blind panel consisting of 40 naïve urine samples, was determined. The 40 naïve samples consisted of 20 samples collected during the current study, but not used in the generation of the classifier, as well as an additional 20 samples that were generated by different mice during a separate study.

The 40 naïve test samples were divided into 2 groups. Group A consisted of 20 samples produced by animals at time points earlier in the disease course than the samples used to generate the classifier. The panel was composed of urine samples representing 8 disease free, 9 Me-7 infected, and 3 Alzheimer diseased mice. The identification of Me-7 infected samples by the 20 feature disease discriminate classifier was 11% sensitive and 91% specific. Specifically, 1 false positive and 8 false negatives (Table 3).

**Table 3:** Classification results applying the 20 protein Disease Discriminant Classifier to 20 naïve samples acquired from earlier in the disease course.

Blind #	Alz	Me-7	Normal
**1**			√
**2**			√
**3**		*	X
**4**		*	X
**5**			√
**6**			√
**7**		*	X
**8**	*		X
**9**	*		X
**10**			√
**11**	X	*	
**12**	*		X
**13**			√
**14**		*	X
**15**		X	*
**16**	X	*	
**17**		*	X
**18**			√
**19**		√	
**20**		*	X

√ indicates correctly classified samples.

X indicates incorrectly classified samples.

* indicates the correct classification for the misclassified samples.

The 20 samples of Group B were produced by animals at points in time where the diseased animals were beginning to exhibits signs of the clinical disease. This put these samples at the same stage of disease progression as the samples used to generate the classifier. The panel was composed of urine samples representing 6 disease free, 7 Me-7 infected and 7 Alzheimer diseased mice. In this instance the identification of Me-7 mice by the 20 protein disease discriminant classifier was 87% sensitive and 93% specific. Specifically, with respect to the identification of the Me-7 infected samples there were 1 false positive and 1 false negative (Table 4). Significantly, the identification of the Alzheimer diseased mice by the 20 protein disease discriminate classifier was also 87% sensitive and 93% specific.

**Table 4:** Classification results applying the 20 protein Disease Discriminant Classifier to 20 naïve samples obtained when the disease stage of the test set mirrored that of the training set.

Blind #	Alz	Me-7	Normal
**1**	√		
**2**	*		X
**3**		√	
**4**			√
**5**	√		
**6**			√
**7**		√	
**8**		√	
**9**		√	
**10**	√		
**11**		*	X
**12**	X		*
**13**		X	*
**14**	√		
**15**	√		
**16**			√
**17**			√
**18**	√		
**19**		√	
**20**		√	

√ indicates correctly classified samples.

X indicates incorrectly classified samples.

* indicates the correct classification for the misclassified samples.

## Discussion

Previously, the differential abundance of a panel of bovine urine proteins was found sufficient to identify BSE infected cattle at the clinical stage of the disease irrespective of the presence of confounding factors such as breed, gender or age [16]. In order to further validate the utilization of urine as the basis of a novel test for TSE disease the present study was conducted. The object was to determine whether a panel of urine based biomarkers, specific for TSE disease as opposed to generic indicators of infection or disease, could be identified.

In order to make this determination urine samples derived from other neurologically diseased, TSE negative animals were required. To meet this requirement we took advantage of the availability of a transgenic murine model of Alzheimer's disease. A prerequisite for the use of murine models of neurodegenerative disease for this project was a demonstration that TSE disease induced changes in the urine proteome occurred in mice as well as cattle. A scrapie specific classifier based on the differential abundance of two proteins, able to discriminate between control and scrapie infected mice when applied to the samples of the training set (≥11 wpi), was 100% (Figure 1). It was expected that the true accuracy of the classifier when applied to an independent data set would be lower. Nonetheless, these experiments demonstrated that, in a mouse model of scrapie, differential protein abundance in urine was modulated by TSE infection.

In the transgenic Alzheimer's disease model the diseased animals and their prescribed controls were not isogenic. This suggested that the consistently high degree of accuracy of the classifiers identified prior to the appearance of disease symptoms might have been indicative of the genetic drift that can occur in inbred mouse colonies in the absence of selective pressure. Sub-strains can be generated in as few as 10 generations (Table 1, http://jaxmice.jax.org/genetichealth/drift.html). However, once the mice had reached 32 weeks of age, where presumably disease specific changes dominated the differential abundance observed in the urine protein profile, an Alzheimer's disease specific classifier with a calculated accuracy of 100% was identified (Figure 3).

When the clinical stage samples and age matched controls of the two neurodegenerative disease models were combined, a disease discriminant classifier was defined that was able to correctly identify all 38 samples. However, the presence of strain influenced features within this classifier was made abundantly apparent by the clear separation of the two different control sample cohorts into different quadrants of a PCA plot (Figure 4). The inability of EDA to distinguish the difference between strain and disease specific classifiers precluded its use to define a disease discriminant classifier between the three groups. Instead the DeCyder software was used to make pairwise comparisons between the four groups. The results of these discriminant analyses were then used to both exclude and select from the initial 169 features of the disease discriminant classifier features a subset of 55 features that remained able to discriminate between the two diseases and the combined controls with a calculated accuracy of 100% (Figures 5 and 6).

Due to the necessity of excising spots in order to identify the associated proteins a reduced disease discriminant classifier based on the 20 easily visualized features on a stained gel were chosen. The excision and mass spectrometry analysis of 11 of the 20 features of the disease discriminant classifier lead to the identification of 22 proteins (Table 2). In addition to their role as putative biomarkers, the 22 proteins can be associated with a variety of physiologic functions. Two are abundant urine proteins, albumin responsible for maintaining osmotic pressure and MUP 1 that slows the release aromatic compounds used for identification and the marking of territory [32]–[34]. Many of the remainder are either associated with the formation of desmosomes [35]–[39] or can be linked to neurodegenerative disease processes [25], [40]–[49]. Further study is needed to confirm whether the differential abundance of these proteins in the urine is indicative of diseases processes occurring in the primary site of TSE biology, the brain, or other organs. For example, possible renal dysfunction was suggested by the presence of napsin A and transthyretin [50], [51] in the urine. The observed significant (p≤0.012) decrease in the glomerular filtration rate indicated that in this mouse model renal dysfunction coincides with the onset of clinical disease. Whether this effect is due to the infection or an indirect consequence of the disease is not known. However, it does indicate that in addition to being disease biomarkers further investigation of the proteins associated with the features may provide insight into pathways and mechanisms related to the observed pathology.

The calculated accuracy of the reduced (20 feature) classifier when applied to the training set was 98.3±3.7% (Figure 6). Independent data sets, of 20 preclinical and 20 clinical samples, not involved in the determination of the classifier parameters, were used to measure the true accuracy. The sensitivity of the reduced classifier when applied to preclinical samples was only 11% (Table 3). The classification of the 8 false negatives as normal supported the suggestion that disease specific changes to the urine proteome only emerge in the late stages of the disease.

The second set of naïve samples, derived from mice of both disease models at the clinical stage of the disease, mirrored the disease stage of the training set. The sensitivity and specificity of the reduced classifier on this set of samples was 87% and 93% respectively (Table 4). Importantly, the classifier not only correctly identified the 12 diseased samples, but placed them into the correct disease category (scrapie or Alzheimer) 100% of the time. This result compares favourably with the current “state of the art” RT-QuIC assay that is in the process of being validated for the diagnoses of human CJD cases where the sample used is cerebral spinal fluid (CSF) [6], [7]. Thus, in the context of a clinical evaluation the differential abundance of urine proteins may be useful in distinguishing TSE from other neurodegenerative diseases.

## Supporting Information

Figure S1 **Diagrammatic representation of raw spot feature data.** A) This is a limited view of the same gel image as Figure 5. The brightness settings have been adjusted to increase the visibility of individual spots that appear saturated in Figure 5. The spots that are truly saturated and excluded from the analysis have grey spot boundaries. B) This is a 3 dimensional representation of the image in A. Note that the saturated spots, again identified by the grey boundary all reach a maximum value where their peaks become flat topped. Three proteins that are part of the disease discriminate classifier and indicated by arrows. Their maximum intensity is much below the level of saturation. C) Is a magnified view of the 3 dimensional representation of the data shown in B to make the individual peaks more visible. The view of peak 776 is partly obscured by the two larger peaks in the foreground of the image. D) The histograms represent the relative average abundance of the spots in the 7 samples produced by 4 scrapie infected animals at 15 and 17 wpi, the 11 samples obtained from 4 Alzheimer's disease model animals at 32,36 and 40 weeks of age and the 20 age matched control samples produced by the 8 corresponding control mice. The error bars indicate the standard deviation. The average abundance of the protein(s) represented by spot 895) in the urine of the three groups were all significantly different from one another. In contrast, the abundance of the protein(s) represented by spots 776 and 786 were similar in the control and scrapie infected animals both of which had significantly higher levels than that observed in the Alzheimer's disease model mice.
(TIF)

Table S1 Glomerular filtration rate calculations.
(XLSX)

Table S2 Statistical analysis of glomerular filtration rate.
(XLSX)
