# Supplementary figures and images for: Urine Proteins Identified by Two-Dimensional Differential Gel Electrophoresis Facilitate the Differential Diagnoses of Scrapie
Source: PLoS One. 2013 May 21;8(5):e64044. doi: 10.1371/journal.pone.0064044 (PMC3660319; doi:10.1371/journal.pone.0064044)

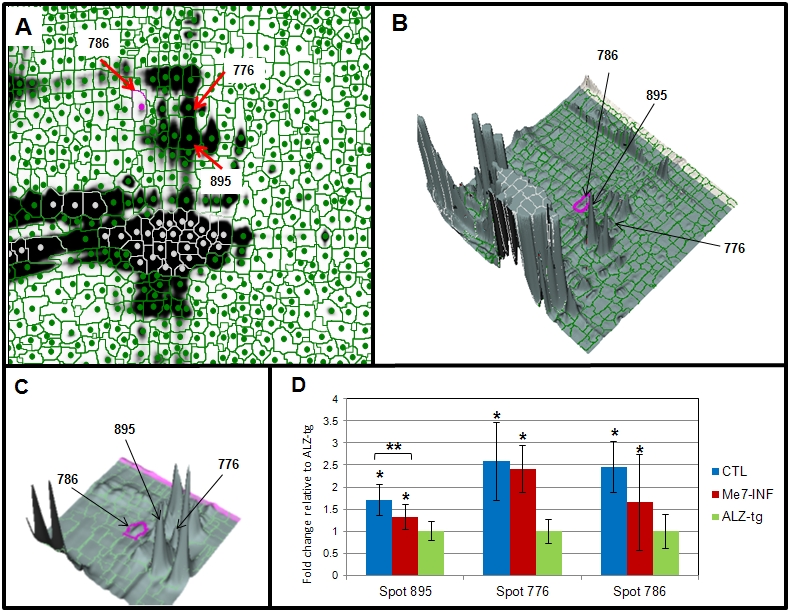

Supplement: Figure S1 — Diagrammatic representation of raw spot feature data. A) This is a limited view of the same gel image as Figure 5. The brightness settings have been adjusted to increase the visibility of individual spots that appear saturated in Figure 5. The spots that are truly saturated and excluded from the analysis have grey spot boundaries. B) This is a 3 dimensional representation of the image in A. Note that the saturated spots, again identified by the grey boundary all reach a maximum value where their peaks become flat topped. Three proteins that are part of the disease discriminate classifier and indicated by arrows. Their maximum intensity is much below the level of saturation. C) Is a magnified view of the 3 dimensional representation of the data shown in B to make the individual peaks more visible. The view of peak 776 is partly obscured by the two larger peaks in the foreground of the image. D) The histograms represent the relative average abundance of the spots in the 7 samples produced by 4 scrapie infected animals at 15 and 17 wpi, the 11 samples obtained from 4 Alzheimer's disease model animals at 32,36 and 40 weeks of age and the 20 age matched control samples produced by the 8 corresponding control mice. The error bars indicate the standard deviation. The average abundance of the protein(s) represented by spot 895) in the urine of the three groups were all significantly different from one another. In contrast, the abundance of the protein(s) represented by spots 776 and 786 were similar in the control and scrapie infected animals both of which had significantly higher levels than that observed in the Alzheimer's disease model mice. (TIF) [file pone.0064044.s001.tif]
